# Supplementary material for: HPLC-Based Purification and Isolation of Potent Anti-HIV and Latency Reversing Daphnane Diterpenes from the Medicinal Plant Gnidia sericocephala (Thymelaeaceae)
Source: Viruses. 2022 Jun 30;14(7):1437. doi: 10.3390/v14071437 (PMC9318819; doi:10.3390/v14071437)
Supplement: Supplementary file 1 [file viruses-14-01437-s001.zip › viruses-1761495-SI.pdf]

HPLC-based purification and isolation of potent anti-HIV and latency reversing daphnane diterpenes from the medicinal plant *Gnidia sericocephala* (*Thymelaeaceae*)

Babalwa Tembeni [1*], Amanda Sciorillo [2*], Luke Invernizzi [1], Thomas Klimkait [3], Lorena Urda [3], Phanankosi Moyo [1], Dashnie Naidoo-Maharaj [1], Nathan Levitties [2], Kwasi Gyampoh [2], Guorui Zu [2], Zhe Yuan [2], Karam Mounzer [4], Siphathimandla Nkabinde[5], Magugu Nkabinde[5], Nceba Gqaleni[6,7,8], Ian Tietjen [2], Luis J. Montaner [2#], and Vinesh Maharaj [1#]

[1] Department of Chemistry, University of Pretoria, South Africa

[2] The Wistar Institute, Philadelphia, United States

[3] Molecular Virology, Department of Biomedicine, University of Basel, Switzerland

[4] Jonathan Lax Immune Disorders Treatment Center, Philadelphia Fight Community Health Centers, Philadelphia, United States

[5]J Uitval, Wasbank 2920, South Africa

[6]Africa Health Research Institute, [7]Discipline of Traditional Medicine, University of KwaZulu-Natal, [8]Faculty of Health Sciences, Durban University of Technology, Durban, South Africa

* These authors contributed equally

# Corresponding authors. V. M. vinesh.maharaj@up.ac.za and L.J.M.: montaner@wistar.org

Babalwa Tembeni (btembeni2@gmail.com)(ORCID: 0000-0001-7126-3447)
Amanda Sciorillo (asciorillo@wistar.org)
Prof Thomas Klimkait (thomas.klimkait@unibas.ch)
Lorena Urda (lorena.urda@unibas.ch)
Luke Invernizzi (lukeinvernizzi@gmail.com) (ORCID: 0000-0003-1095-365X)
Dr. Phanankosi Moyo (phanankosimoyo@gmail.com) (ORCID: 0000-0001-7692-3256)
Dashnie Naidoo-Maharaj (Dashnie.naidoo@up.ac.za)
Nathan Levitties (nlevitties@gmail.com)
Kwasi Gyampoh (kgyampoh@wistar.org)
Guorui Zu (gzu@wistar.org)

Zhe Yuan (zyuan@wistar.org)
Karam Mounzer (mounzerk@fight.org)
Siphathimandla Nkabinde (magugun@webmail.co.za)
Magugu Nkabinde (magugun@webmail.co.za)
Prof Nceba Gqaleni (nceba.gqaleni@ahri.org)
Prof. Ian Tietjen (itietjen@wistar.org) (ORCID: 0000-0002-8991-6490)
Prof. Luis J. Montaner (montaner@wistar.org) (ORCID: 0000-0001-5799-6759)
Prof. Vinesh J. Maharaj (vinesh.maharaj@up.ac.za) (ORCID: 0000-0002-6512-304X)

Table S1: Anti-HIV replication activity of the positive control efavirenz using the *in vitro deCIPhR* assay.

% Inhibition and cytotoxicity of the positive control				
**Efavirenz**	**4300 nM**	**2000 nM**	**930 nM**	**430 nM**
%Inhibition	100.0±0.0	106.6±24.1	110.7±22.1	43.9±41.9
%Cytotoxicity	NC	NC	NC	NC

NC – not cytotoxic

Table S2: Anti-HIV replication activity of *G. sericocephala* root extracts using the *in vitro deCIPhR* assay.

**Extracts**	**% Inhibition of HIV replication**					
	**0.8 µg/ml**	**2.5 µg/ml**	**7.4 µg/ml**	**22 µg/ml**	**67 µg/ml**	**200 µg/ml**
n-Hexane	77.0±5.3	124±2.2	101.6±4.0	43.7±8.3	NA	38.4±47.0
Dichloromethane	113.8±1.8	99.7±4.4	82.6±4.2	87.5±5.8	116.6±3.0	116.7±2.2
Ethyl acetate	18.1±8.4	1.5±17.9	103.6±7.3	131.6±5.6	137.9±6.4	140.2±4.0
Methanol	NA	NA	NA	89.9±12.0	138.0±5.5	134.8±5.6

NA – not active

Table S3: Cytotoxicity of *G. sericocephala* root extracts using the *in vitro deCIPhR* assay.

**Extracts**	**% Cytotoxicity of *G.sericocephala* extracts**					
	**0.8 µg/ml**	**2.5 µg/ml**	**7.4 µg/ml**	**22 µg/ml**	**67 µg/ml**	**200 µg/ml**
n-Hexane	NA	19.1±0.3	11.3±2.6	4.5±5.9	NA	19.1±3.7
Dichloromethane	NA	10.2±1.4	11.6±12.8	42.0±12.2	94.1±3.5	102.5±0.2
Ethyl acetate	NA	NA	6.4±8.2	30.2±4.6	50.4±4.7	101.5±1.7
Methanol	NA	NA	NA	NA	65.8±8.3	101.1±1.3

NA – not active

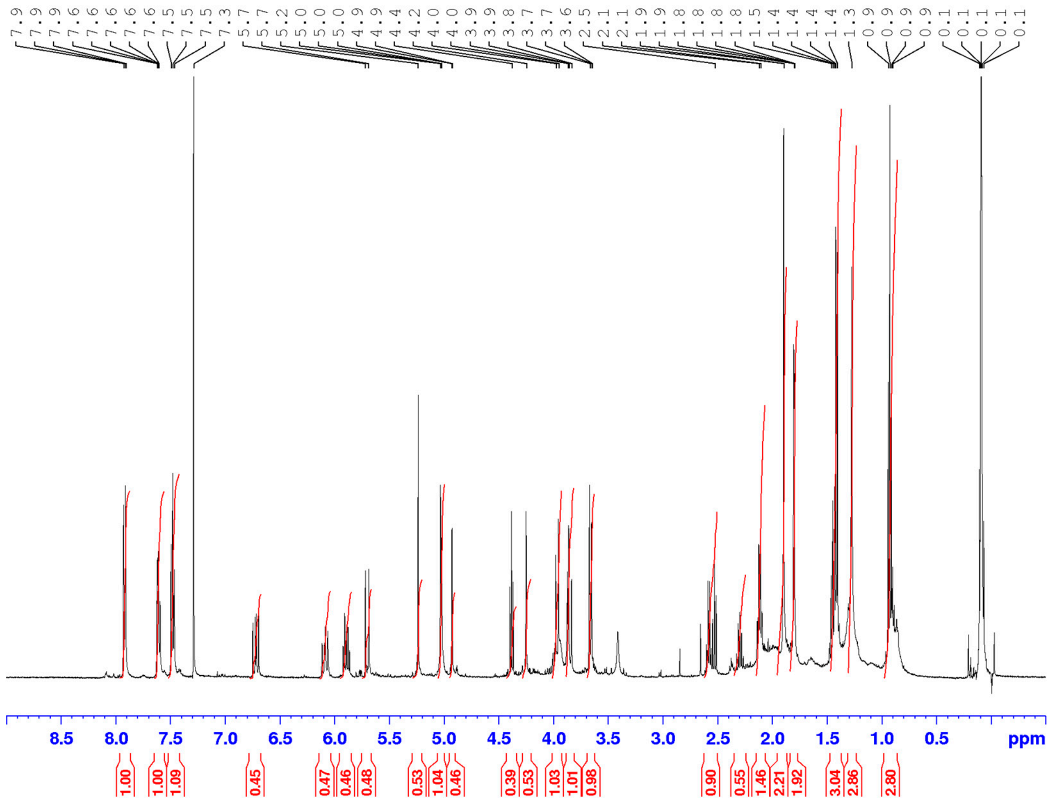

Figure S1: ^{1}H NMR data of yuanhuacine A (**1**), acquired on a Bruker Avance III HD 500 MHz NMR spectrophotometer with Prodigy Probe, the compound dissolved in deuterated chloroform ($CDCl_3$).

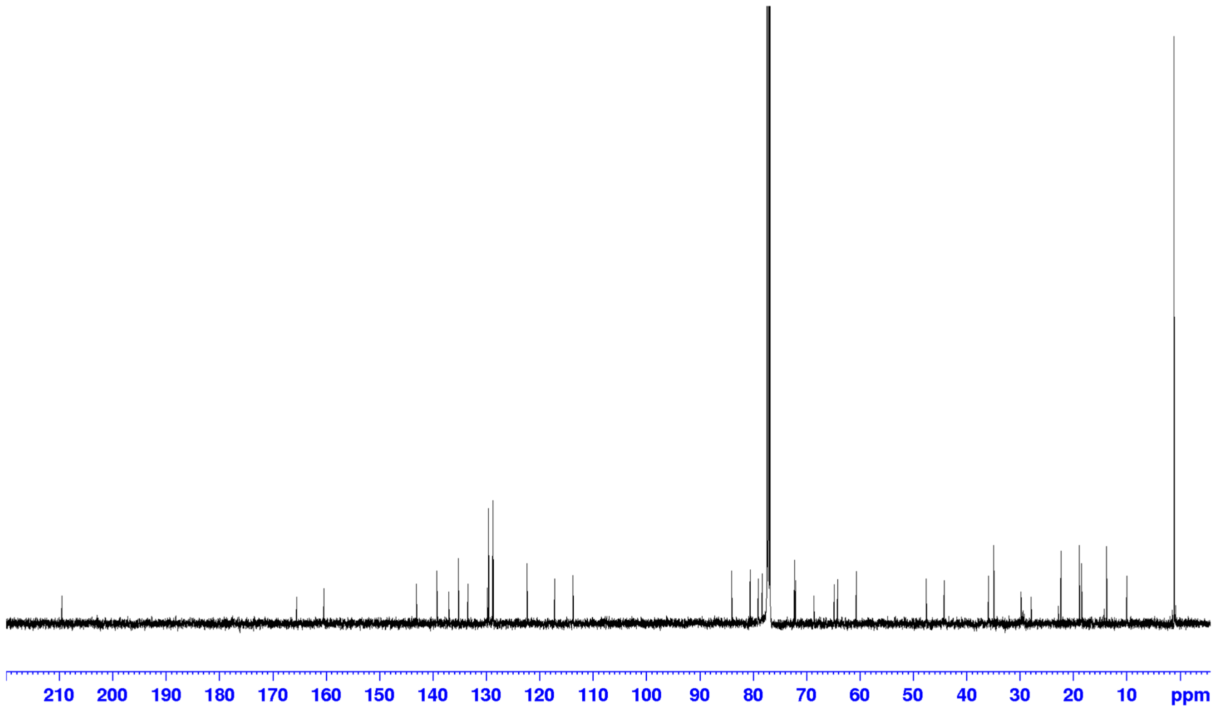

Figure S2: ^{13}C NMR data of yuanhuacine A (**1**), acquired on a Bruker Avance III HD 500 MHz NMR spectrophotometer with Prodigy Probe, the compound dissolved in deuterated chloroform ($CDCl_3$).

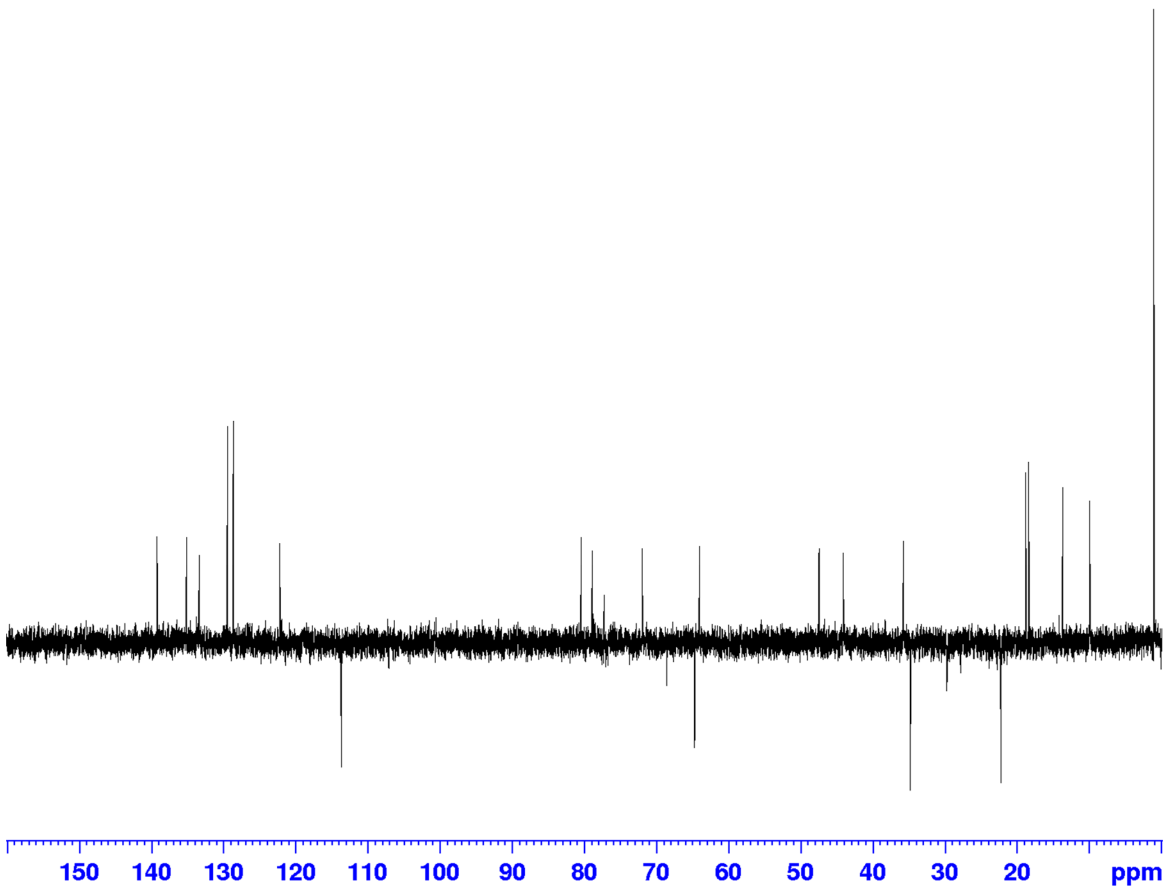

Figure S3: The DEBT NMR data of yuanhuacine A (**1)**, acquired on a Bruker Avance III HD 500 MHz NMR spectrophotometer with Prodigy Probe, the compound dissolved in deuterated chloroform ($CDCl_3$).

Observational studies S1

Unpublished data on the Effects of Product Nkabinde on viral load and CD4 counts of HIV+ adults: an observational study (unpublished)

M J Stewart, ^W Nkabinde, *L Morris, *N Taylor, T Snyman, and #N Gqaleni

Department of Chemical Pathology, University of the Witwatersrand, ^Ungangezulu Indigenous Remedies, * South African National Institute of Communicable Diseases, and #Traditional Medicine Laboratory, University of KwaZulu-Natal

These studies consisted of retrospective analyses of CD4 and viral load tests carried out over a period of 4 years on patients who had medical aid (insurance) at the time. Most of the patients treated over the past four years with this remedy were not covered by medical aid and therefore no laboratory studies could be obtained for analysis. In 35 cases, patients with medical aid, laboratory estimates of haematological parameters, (including CD4 counts) and viral load were carried out. In 8 of these cases the data was incomplete.

In 26 of the 27 cases for which results were available, the laboratories used were accredited by the South African National Accreditation System (SANAS). Patients had been treated with the remedy for periods ranging from one month to 22 months. Results from patients in whom there was suspicion that ARVs may have been prescribed by a conventional clinic were discarded.

Results of analysis

Viral loads

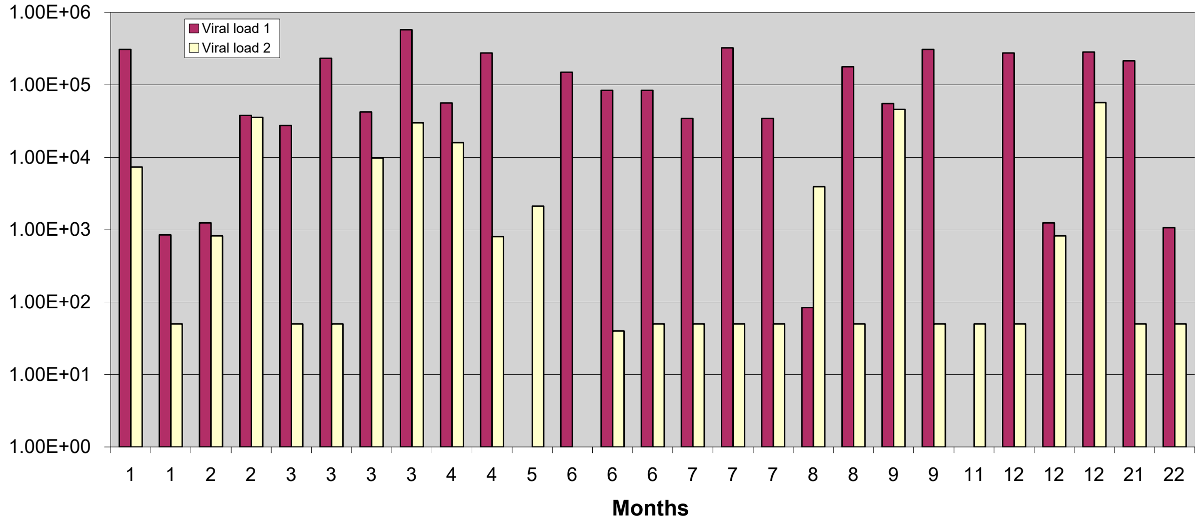

Percentage reduction of viral loads

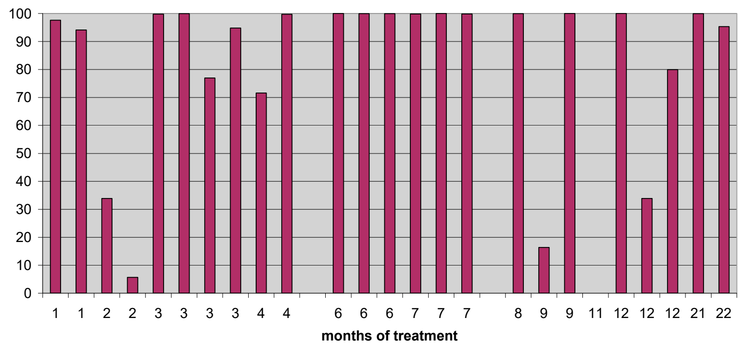

CD4 counts

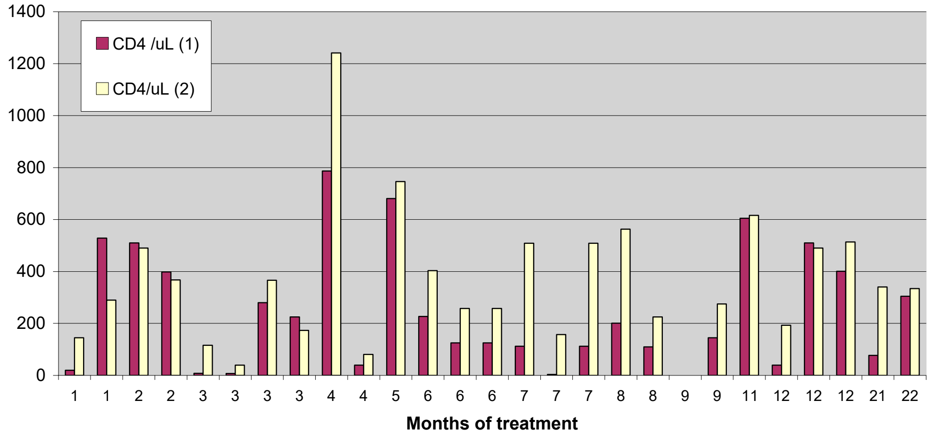

Percentage change in CD4 counts

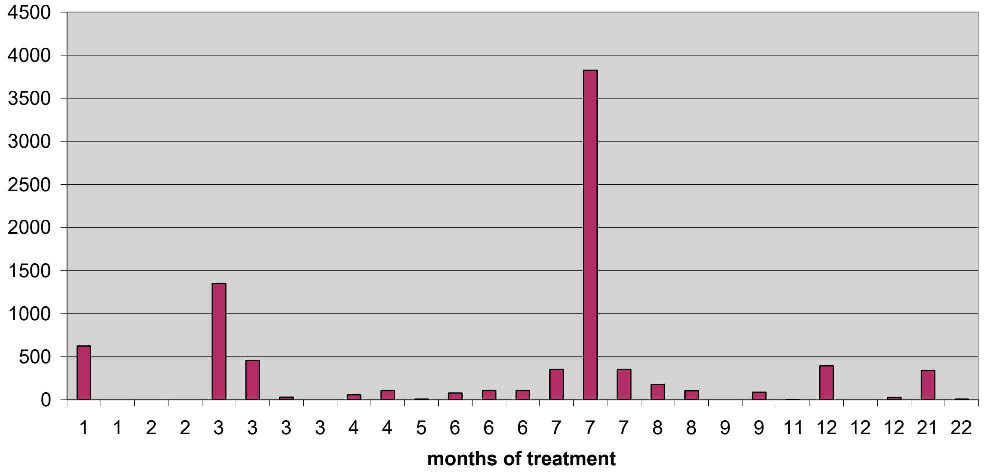

Summary of in vivo analysis

It should be noted that the data are not clean, in that they are retrospective and also that there is no objective evidence that patients were not also taking other medication. The results indicated that the viral load was reduced in 24 of the 27 patients. In 13 of these it was reduced to less than 50 copies there was a rise in CD4 counts in 23 of the 27 patients over periods of 1-22 months of treatment.
